# Diverging opinions about shared decisions

**DOI:** 10.1007/s12471-014-0569-1

**Published:** 2014-06-19

**Authors:** C. A. Swenne

**Affiliations:** Department of Cardiology, Leiden University Medical Center, PO Box 9600, 2300 RC Leiden, the Netherlands

Shared decision making is an emerging physician-patient interaction model for clinical practice [1]. Essentially, shared decision making implies that both the physician and the patient contribute to and bear responsibility for the clinical decision to be taken. It offers an alternative for the paternalistic model, in which it is the physician who informs the patient and proposes the decision to be made. Shared decision making emphasizes patient’s autonomy and recognizes the argumentation and preferences of the informed patient as valid elements in the decision process. Thus, it can dramatically influence the physician-patient relationship. E.g., in a given case, the choice for doing nothing as an alternative for pharmacotherapy could be considered as an acceptable outcome of the shared decision making process, while it could be considered as disobedient behavior of the patient in the paternalistic model.

Clinical practice guidelines usually define a single best option in a given case [2]. Seen from this perspective, guidelines leave little freedom for the patient and reinforce the paternalistic model rather than shared decision making. Moreover, the shared decision making model is not universally preferred; numerous situations can be mentioned in which either patient or physician would prefer the paternalistic model [1]. Shared decision making is, however, particularly important when trade-offs between options strongly depend on individual preferences. This includes recommendations within guidelines for which the evidence is scarce or conflicting or for which there is more than one relevant treatment option that different individuals may value differently [2]. This explains why more and more recommendations for shared decision making appear in new guidelines. One of these guidelines is the 2012 version of the Guidelines on the Management of Valvular Heart Disease, by the Joint Task Force on the Management of Valvular Heart Disease of the European Society of Cardiology (ESC) and the European Association for Cardio-Thoracic Surgery (EACTS) [3], that reads, on page S9: “Finally, a decision should be reached through the process of shared decision-making, first by a multidisciplinary ‘heart team’ discussion, then by informing the patient thoroughly, and finally by deciding with the patient and family which treatment option is optimal”. Also the new 2014 AHA/ACC Guideline for the Management of Patients With Valvular Heart Disease [4] mentions shared decision making several times. Amongst others, this guideline gives the following class I, level of evidence C recommendation: “The choice of valve intervention, that is, repair or replacement, as well as type of prosthetic heart valve, should be a shared decisionmaking process that accounts for the patient’s values and preferences, with full disclosure of the indications for and risks of anticoagulant therapy and the potential need for and risk of reoperation.” Also, in the AHA/ACC guideline, discussion of individual cases in a multidisciplinary ‘heart valve team’ is considered essential.

Indeed, extra complexity in shared decision making can arise if multidisciplinary expertise is involved, e.g., cardiology and thoracic sugery in the case of cardiac valve disease. In the current issue of the Netherlands Heart Journal, Korteland and colleagues [5] present a survey among Dutch cardiologists and cardiac surgeons regarding their opinion on decision making in prosthetic aortic valve selection. A total of 117 physicians participated, 54 cardiothoracic surgeons (11 in training) and 63 cardiologists (7 in training), representing 38 % and 6 % of the Dutch cardiothoracic/cardiologist population, respectively. Most respondents agreed that patients should be involved in decision making, with surgeons leaning more toward patient involvement than cardiologists. Most respondents found that patients and doctors should decide together, with cardiologists leaning more toward taking the lead than surgeons. Physicians working in a centre with cardiac surgery were more inclined to decide together with the patient while physicians working in a centre without a cardiac surgery program more often preferred to take the lead in decision making.

Shared decision making may not fit all forms of clinical practice, but it seems an appropriate approach in valve intervention, as underscored by the guidelines [3, 4]. It is not sure how much the study results of Korteland and colleagues [5] were influenced by selection bias. Assuming that selection bias is limited, their study suggests that shared decision making in prosthetic aortic valve selection is quite commonly performed in the Netherlands. The difference between the cardiologists’ and surgeons’ responses to the questionnaire remains puzzling. At the same time it is one main *raison d*'*être* for the multidisciplinary heart team as recommended in the guidelines [3, 4].

## References

[1] Rodriguez-Osorio CA, Dominguez-Cherit G (2008). Medical decision making: paternalism versus patient-centered (autonomous) care. Curr Opin Crit Care.

[2] Van der Weijden T, Pieterse AH, Koelewijn-van Loon MS (2013). How can clinical practice guidelines be adapted to facilitate shared decision making? A qualitative key-informant study. BMJ Qual Saf.

[3] Vahanian A, Alfieri O, Andreotti F (2012). Guidelines on the management of valvular heart disease (version 2012). Eur Heart J.

[4] Nishimura RA, Otto CM, Bonow RO (2014). 2014 AHA/ACC Guideline for the management of patients with valvular heart disease: a report of the American College of Cardiology/American Heart Association Task Force on Practice Guidelines. J Am Coll Cardiol.

[5] Korteland NM, Kluin J, Klautz RJM, et al. Cardiologist and cardiac surgeon view on decision-making in prosthetic aortic valve selection: does profession matter? Neth Heart J 2014; this issue. doi 10.1007/s12471-014-0564-6 10.1007/s12471-014-0564-6 PMC4099434 24915773

